# Sucrose monolaurate production from lauric acid through a two-stage process

**DOI:** 10.1038/s41598-023-38461-7

**Published:** 2023-07-11

**Authors:** Tiprawee Tongtummachat, Attasak Jaree, Amaraporn Kaewchada, Boonyaporn Sunorakum, Panalee Ignacio, Nattee Akkarawatkhoosith

**Affiliations:** 1 Bio-Based Chemical and Biofuel Engineering Laboratory, Department of Chemical Engineering, Faculty of Engineering, Mahidol University, 25/25 Phuttamonthon 4 Road, Nakhon Pathom, 73170 Thailand; 2 Department of Chemical Engineering, Faculty of Engineering, Kasetsart University, Chatuchak, Bangkok, 10900 Thailand; 3 Department of Agro-Industrial, Food and Environmental Technology, King Mongkut’s University of Technology North Bangkok, Pracharat 1 Road, Wongsawang, Bansue, Bangkok, 10800 Thailand

**Keywords:** Chemical engineering, Chemical synthesis, Synthetic chemistry methodology, Heterogeneous catalysis

## Abstract

This work represented the first step toward pioneering the use of a two-stage process for sucrose monolaurate (sucrose ester) production from lauric acid with high productivity and selectivity. In the first stage, lauric acid was firstly converted into methyl laurate via esterification, followed by the transesterification of methyl laurate into sucrose ester in the second stage. In this research, the first stage of process was primarily focused and thoroughly evaluated. Methyl laurate was continuously produced via lauric acid and methanol in a mini fixed-bed reactor. Amberlyst 15 was used as a catalyst. The operating variables were thoroughly investigated and optimized. The optimal condition to achieve 98 wt% yield (99% purity) was as follows: temperature of 110 °C, residence time of 5 min, and feed concentration of 94 g/L. High catalytic stability was observed over the time-on-stream of 30 h. This process provided good productivity compared to the other processes. The methyl laurate obtained from the first stage could be used as a raw material for the second stage to produce sucrose ester, which was demonstrated experimentally. The high selectivity of 95% of sucrose monolaurate was obtained. The continuous production of sucrose ester from lauric acid could be achieved.

## Introduction

Sugar ester (sugar-based fatty acid ester; SE), consisting of hydrophilic (sugar) and lipophilic (fatty acid) groups, is a non-ionic, non-toxic, and biodegradable surfactant. Sugar ester has been attractively considered one of the outstanding bio-based compounds used in food, cosmetic, and pharmaceutical applications^1^. However, the attention of sugar ester has been hindered by its price. For example, sugar ester price was about 2–10 $/kg (2017)^2^, while the prices of general and non-bio-based surfactants (such as alkylphenol ethoxylates: APEs) was only 0.9–1.8 $/kg (2017)^2^. One of the main barriers is the insufficient sugar ester production performance (low production capacity and product purity), which must be developed.

**Figure 1:**
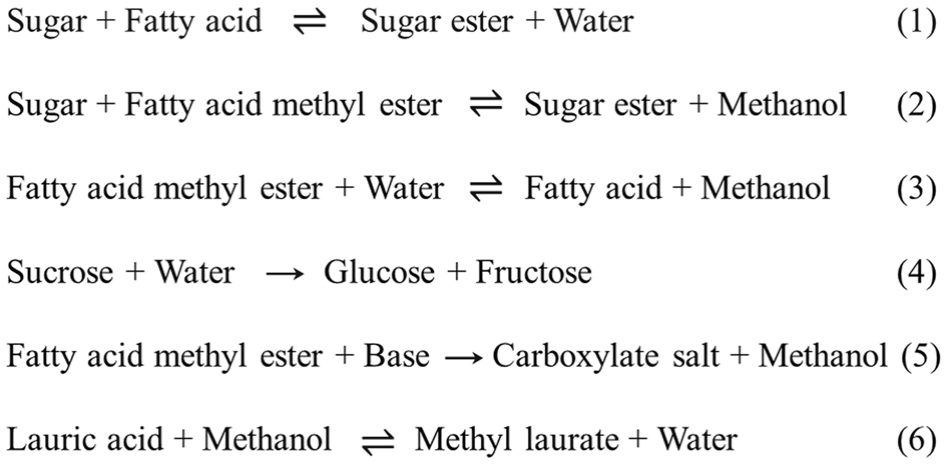
Chemical reactions involved in the production of sucrose monolaurate.

There are two conventional routes for sugar ester production. The first route is called the esterification reaction [see Fig. 1 (reaction (1))]. Sugar is reacted with free fatty acid under a homogenous acid catalyst to produce sugar ester and water. The second route involves the transesterification of fatty acid ester and sugar in the presence of a homogenous base catalyst [see Fig. 1 (reaction (2))]. Both routes are generally carried out under reduced pressure (< 3 kPa) and anhydrous conditions in a batch process^3,4^. The major challenge is the low sugar monoester yield/selectivity due to the side reactions (hydrolysis and saponification [see Fig. 1 (reactions (3)–(5))]. Mass transfer limitation caused by an incompatibility between reactants is another issue that needs to be resolved. Several organic solvents have been searched to substitute for water in order to prevent side reactions and to facilitate mass transfer (dissolving both reactants as a monophasic system). Dimethyl formamide (DMF) and dimethyl sulfoxide (DMSO) are commonly used as solvent. Unfortunately, their solubility limits the maximum loading of reactants^5^. Recently, the solvent-free system has been developed and implemented to avoid the use of solvent, cutting down the cost and simplifying the downstream processing^6^. Although the yield was improved, an elevated temperature (130–180 °C) was required to melt the sugar and to accelerate the reactions^7^. The decrease in the yield due to the decomposition of sugar was also a concern. The recycling of catalysts and catalyst separation are the critical factors that need to be considered for economic sustainability. Hence, a promising heterogeneous-based method that can enhance the production capacity (via continuous process) and the yield of sugar ester needs further investigation.

Among sugar esters, sucrose monoesters are the most desirable compound for pharmaceutical applications, particularly for drug delivery enhancement^1^. For the purpose of absorption and penetration improvement, sucrose monolaurate (C_12_ fatty acid) is one of the promising candidates for future natural water-soluble surfactant. However, the commercial production of this compound has been facing challenges, particularly in achieving high sucrose monoester yield^6^. It is difficult to control the degree of substitution of sucrose since a sucrose molecule contains a reactivity of the eight hydroxyl groups. Therefore, the novel production process of sucrose monolaurate was developed and proposed in this work.

Mini fixed-bed reactor technology is one of the efficient continuous reactors that can offer high rates of heat and mass transfer, demonstrated by our research group for various applications^8,9^. The outstanding production performances were presented when compared to the other production technologies. For example, in the 5-Hydroxymethylfurfural (5-HMF) application, the relatively high yield of 5-HMF produced in a mini-fixed bed reactor was achieved compared to those of other reactors^9^. However, this technology has never been implemented for the sugar ester production. Hence, in this work, the mini fixed-bed reactor was applied to explore its effectiveness to overcome the technical challenges for the production of sugar ester.

**Figure 2:**
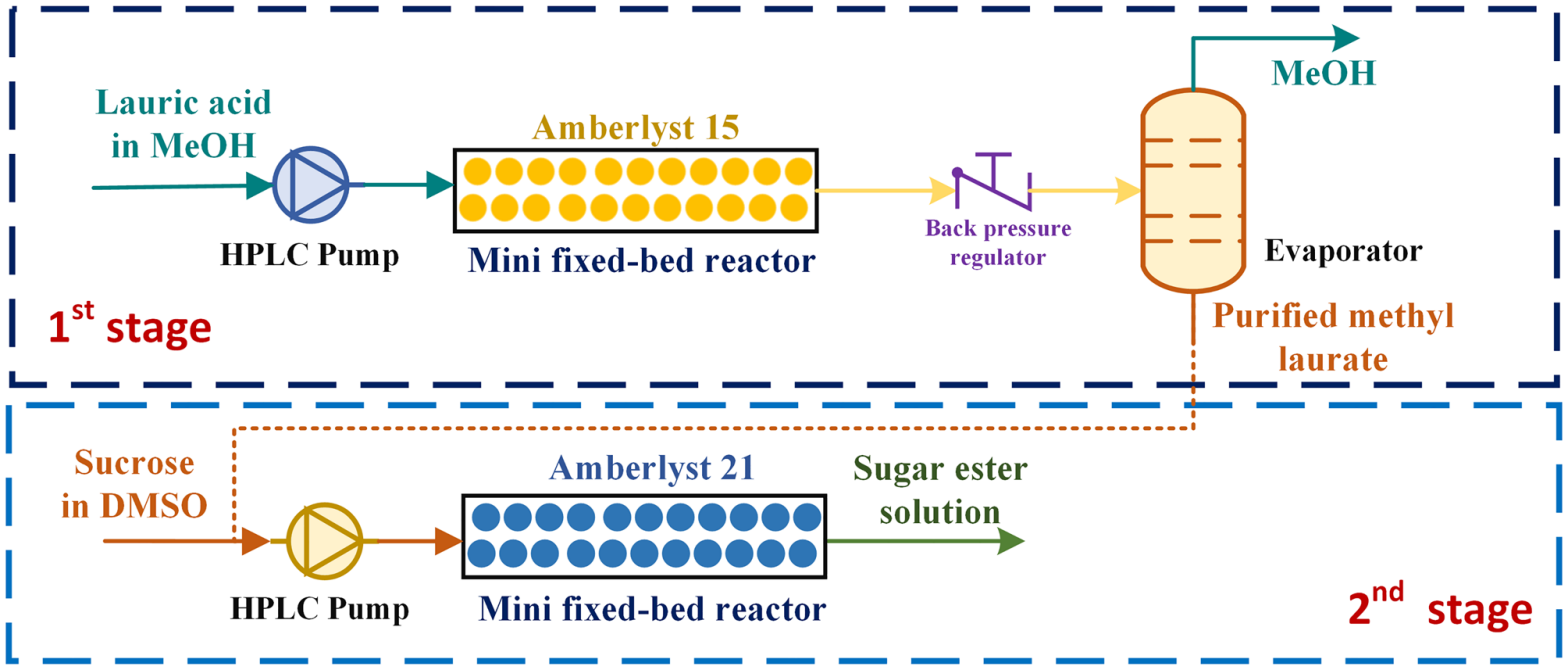
Continuous production of sucrose monolaurate from lauric acid through a two-stage process (Figure was drawn with Microsoft Professional Visio, 2019).

This work proposed the two-stage process for the production of sucrose monolaurate, which has rarely been studied, to enhance production capacity and efficiency. This production process could offer a small degree of side reactions compared to that of the direct production route. The level of substitution could be easily controlled by this proposed method. In addition, the inefficient heat and mass transfers could be mitigated via the use of mini fixed-bed technology. The main goal of this research was to focus on the development of the first-stage process. In this production route (see Fig. 2), the lauric acid was firstly converted into lauric acid methyl ester (methyl laurate) via esterification. The operating variables affecting the product yield were investigated and optimized. The production performance of our proposed process was compared to the other techniques from the literature. The second stage of the process was also performed to confirm the feasibility of using the two-stage process for production of sucrose monolaurate.

## Materials and methods

### Materials

For the first stage of the process, lauric acid (≥ 98%) and methanol (99.9%) were purchased from Thermo Scientific and RCI Labscan, respectively. Amberlyst 15 was obtained from Alfa Aesar and used without any modification. For the second stage of the process, sucrose was purchased from Merck and DMSO (≥ 99%) was obtained from Loba Chemical. Amberlyst 21 from Alfa Aesar was used as a catalyst without any pretreatment. Methyl laurate (≥ 98%) and sucrose monolaurate (98%) were obtained from Sigma-Aldrich for analytical purposes.

### Methyl laurate production (the first stage process)

In the first stage of sugar ester production, methyl laurate was continuously produced via esterification of lauric acid and methanol in a mini-fixed bed reactor (inside diameter: 4.6 mm, reactor volume: 3.3 mL) [see Fig. 1 (reaction (6))]. In this process, lauric acid solution (47 to 330 g/L) was fed into the mini-fixed bed reactor, where the Amberlyst 15 (2.7 g) was packed and used as catalyst to accelerate the reaction. Amberlyst 15 is one of the effective catalysts for esterification reaction^10,11^. High catalytic stability was the highlight of this catalyst^9^. The volumetric flow rate was adjusted according to the residence time studied [see Eq. (1)]. The bed porosity of our reactor was 0.67, which was conducted based on our previous work^8^. The temperature and pressure were maintained by the convection oven and back pressure regulator, respectively. The product outlet was purified using deionized water and a centrifugal separator to separate the solvent. The product was washed twice to obtain high-purity product. The purified product was analyzed using by High-performance liquid chromatography (HPLC) technique to determine the amount of methyl laurate. This data was used to evaluate the production performance [see Eqs. (2) and (3)]. The purified methyl laurate was then used as raw material for sugar ester production (second stage of the process). A schematic view of the experimental setup is shown in Fig. 2.

1 $${\text{Residence}}\;{\text{time}}\;\left( {{\text{min}}} \right) \, = {\text{reactor}}\;{\text{volume }} \times {\text{ bed }}\;{\text{porosity}}/{\text{volumetric }}\;{\text{flowrate}}$$

2 $${\text{Methyl }}\;{\text{laurate }}\;{\text{yield }}\left( {{\text{yield}}, \, \% } \right) = \, \left( {{\text{mole}}\;{\text{ of}}\;{\text{ methyl }}\;{\text{laurate}}/{\text{mole}}\;{\text{ of}}\;{\text{ lauric }}\;{\text{acid}}} \right) \, \times { 1}00\%$$

3 $${\text{Productivity }}\;\left( {{\text{P}}_{0} , \, \;{\text{g}}_{{{\text{product}}}} /{\text{ g}}_{{{\text{cat}}}} - {\text{h}}} \right) \, = {\text{gram}}\;{\text{ of}}\;{\text{ desired }}\;{\text{product}}/\left( {{\text{gram}}\;{\text{ of}}\;{\text{ catalyst }}\;{\text{used }} \times {\text{ residence}}\;{\text{ time}}} \right)$$

### Sucrose ester production (the second stage process)

Methyl laurate obtained from the first stage (or pure methyl laurate) and sucrose were mixed in DMSO to obtain a homogeneous solution. The ratio was adjusted depending on the feed concentration (5 g/L) and the molar ratio of sucrose to methyl laurate (0.63:1). The solution was then fed into the mini fixed-bed reactor packed with Amberlyst 21 (1.7 g). Note that, the literature suggested that base catalyst was required for the high-performance sucrose ester production^3^. Amberlyst 21 has been extensively used as a promising catalyst for base catalyzed reactions^9^; however, this catalyst has never been studied for sugar ester application. The reaction temperature of 120 °C and ambient pressure were held constant. The outlet stream was collected for product analysis. The schematic diagram of continuous two-stage sucrose ester production is shown in Fig. 2. The productivity, yield and selectivity of sucrose monolaurate were calculated using Eqs. (4) to (5), respectively.

4 $${\text{Sucrose }}\;{\text{mono }}\;{\text{laurate}}\;{\text{ yield}}\;\left( \% \right) \, = \, ({\text{mole}}\;{\text{ of}}\;{\text{sucrose}}\;{\text{ mono }}\;{\text{laurate}}/{\text{mole }}\;{\text{of}}\;{\text{ sucrose}}) \, \times { 1}00\%$$

5 $${\text{Sucrose }}\;{\text{mono }}\;{\text{laurate}}\;{\text{ selectivity}}\left( \% \right) \, = \, ({\text{yield}}\;{\text{ of}}\;{\text{sucrose }}\;{\text{mono }}\;{\text{laurate}}/{\text{conversion}}) \, \times { 1}00\%$$

### Product analyst

The components of sucrose esters (mono-, di-, and triester of sucrose) were analyzed by HPLC (Nexera series, Shimadzu) equipped with a refractive index detector (RI; RID-20A, Shimadzu). The analysis was conducted on the ACE Excel C_18_ column (250 mm × 4.6 mm, 5 µm particle size) at 40 °C. The mobile phase was 85:15 (v/v) of methanol and DI water and the volumetric flow rate was 1 mL/min. The sample was analyzed without any treatment and the injection volume was 10 µL. Lauric acid and methyl laurate were quantified by the same method except that the mobile phase was a mixture of acetone and acetonitrile (70:30 v/v). Similarly, sugar compounds, including sucrose, glucose, and fructose were analyzed using ACE excel 5 NH_2_ (250 mm × 4.6 mm × 5 μm) and a mixture of DI and acetonitrile (82:12 v/v) as mobile phase. The analytical standards were used to identify and quantify these compounds.

## Results and discussion

### Main and interaction effects of process variables on methyl laurate production performance

**Figure 3:**
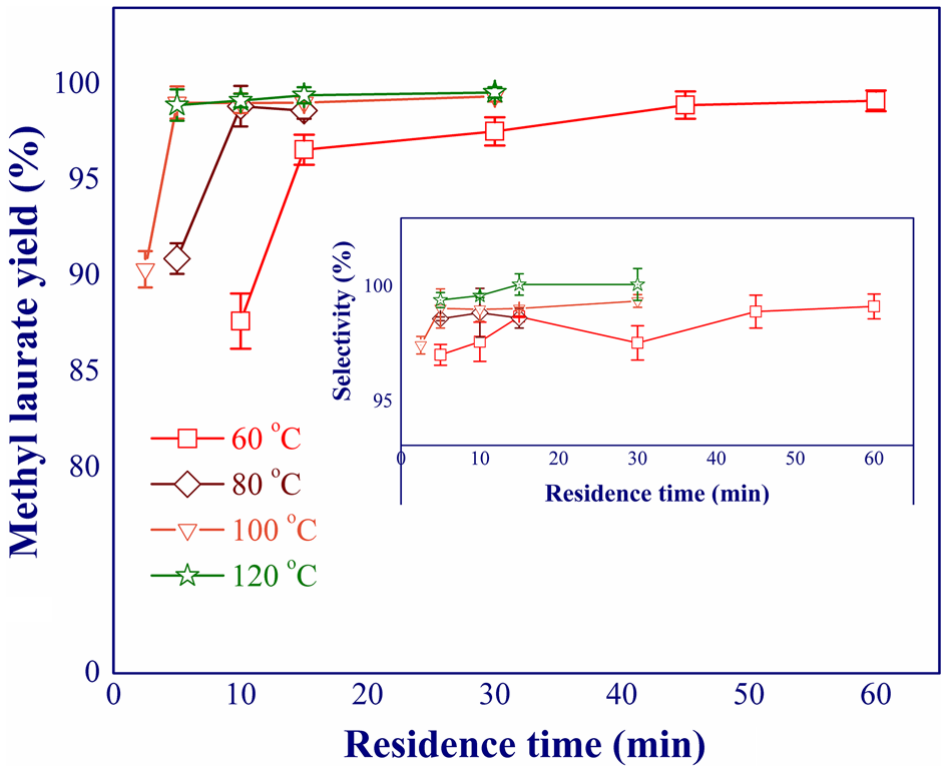
Effect of reaction temperature on methyl laurate yield and selectivity.

In this work, the continuous production of methyl laurate from lauric acid was focused and carried out in a mini fixed-bed reactor packed with Amberlyst 15. Three significant process variables, including reaction temperature (60–120 °C), residence time (2.5–60 min), and concentration of lauric acid (47–329 g/L), were investigated and identified for process optimization. The yield and selectivity of methyl laurate were selected for evaluating the production performance. The first variable was reaction temperature which was assessed under various residence times. The feed concentration of 47 g/L was kept constant (methanol-to-lauric acid molar ratio of 100:1). As shown in Fig. 3, a complete esterification of lauric acid and methanol was achieved at the reaction temperature higher than 100 °C. The yield of more than 99% was achieved with the residence time of 5 min. This revealed that the rate-determining step was mainly dominated by the intrinsic kinetics rather than the mass transports (external/internal mass transfers between solid catalyst and reactants). This was attributed to the facilitated mass transfer using a mini fixed-bed reactor (short diffusion distance). The pore size of the catalyst (Amberlyst 15) was around 33.8 nm, promoting the accessibility and diffusion of the reacting molecules to the catalyst's active sites. The selectivity of 99% indicated that side reaction was not significant under these conditions. A large amount of methanol could inhibit the undesired reactions^12^. However, the side reaction(s) could be detected when using strong acid homogeneous catalysts (such as H_2_SO_4_) at high temperatures. For example, the undesired reaction was reported at temperatures exceeding 90 °C for the esterification reaction^13^. In this research, the reaction temperature of 100 and 120 °C were chosen for further investigation.

**Figure 4:**
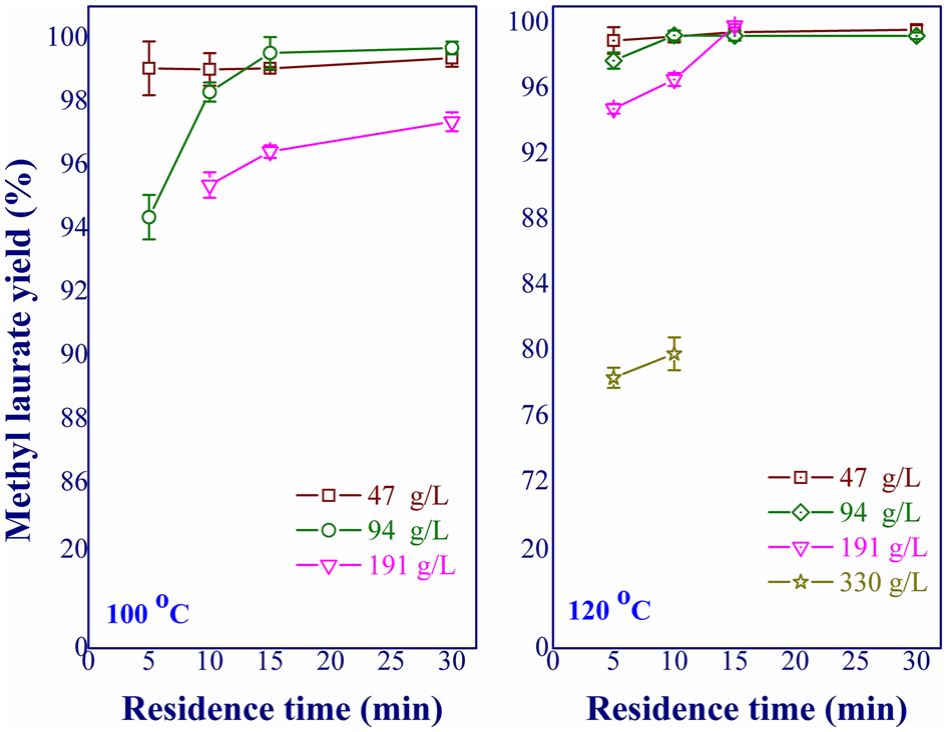
Effect of feed concentration on methyl laurate yield and selectivity.

The following variable was the feed concentration, studied in the range of 47–330 g/L (equivalent to the molar ratio of 100:1 to 12:1). The residence time between 5 and 30 min was focused. The results are shown in Fig. 4. Conceivably, higher reaction temperature and prolonged reaction time were required to obtain high yield when high feed concentration was used. For the feed concentration lower than 191 g/L, complete conversion of lauric acid was achieved under the relatively short residence time (≤ 15 min) and low reaction temperature (≤ 120 °C). Increasing the feed concentration greater than 191 g/L was possible; however, the economic feasibility might be compromised by the requirements of relatively high temperature and prolonged residence time. Hence, the appropriate feed concentration for the cost-effective process was between 94 and 191 g/L. Based on the results, the ranges of variables for the optimization analysis are summarized in Table 1.

**Table 1:** Process variables, range of variables, and constraints for the optimization analysis.

Variables	Symbol	Levels			Constraints
		− 1	0	1	
Independent variables					
Lauric acid concentration (g/L)	X_1_	94	161	191	In the range
Reaction temperature (^o^C)	X_2_	100	110	120	In the range
Residence time (min)	X_3_	5	10	15	In the range
Dependent variable					
Methyl laurate yield (%)	Y				Optimize

**Figure 5:**
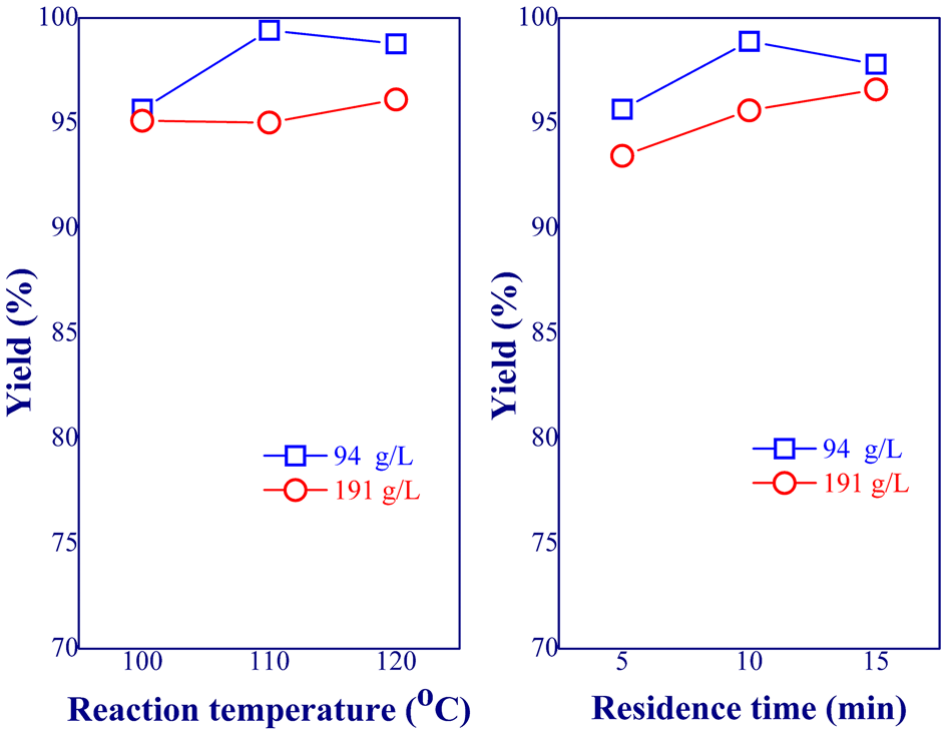
Interaction effects of process variable pairs on methyl laurate yield; *T* temperature, *RT* residence time.

The interaction effects were evaluated based on the analysis of variance (ANOVA) method. Two interesting process variable pairs, temperature-concentration and residence time-concentration, were assessed. The unassessed variables were kept constant at the medium range (level 0) (see Table 1). As shown in Fig. 5, the interaction effects of both process variable pairs were evident, indicated by the difference in the slope of the lines. For the temperature-concentration interaction at low-concentration level, increasing the reaction temperature resulted in the rise of methyl laurate yield before leveling off (> 110 °C). This was due to the complete transesterification reaction. However, at high-concentration level, the temperature higher than 120 °C was required to complete the reaction due to the excess reactant amount. The result was in line with the work of Liu et al.^14^, who studied the esterification of oleic acid with ethanol over organic phosphonic acid/NaY catalyst. For the second pair, the longer residence time was needed to achieve the full conversion when the feed was at high-concentration level. Similar behavior was also reported in the work of Han et al.^15^.

### Optimization of methyl laurate production

This optimization in this work was performed based on the response surface methodology (RSM) coupled with the Box–Behnken design (BBD). The experimental design of three main variables with three levels for each variable is shown in Table 1. The yield of methyl laurate was selected as the response. Note that all experiments were performed in a randomized order with two replications.

The correlation between process variables and the yield of methyl laurate is expressed in the form of a polynomial regression equation, as shown in Eq. (6). The optimal conditions were the reaction temperature of 110 °C, residence time of 5 min, and feed concentration of 94 g/L. The yield and purity of methyl laurate were 98% and 99%, respectively.

6 $${\text{Yield }}\;{\text{of}}\;{\text{ methyl}}\;{\text{ laurate }} = { 167}.{54}0 \, + \, - 0.0{\text{366X}}_{{1}} + \, 0.0{\text{625X}}_{{2}} + \, 0.0{\text{93X}}_{{3}} + {2}.{\text{488x1}}0^{{ - {6}}} {\text{X}}_{{1}}^{{2}} - {2}.{781} \times {1}0^{{ - {5}}} {\text{X}}_{{2}}^{{2}} - 0.00{\text{12X}}_{{3}}^{{2}} + { 7}.{5833} \times {1}0^{{ - {7}}} {\text{X}}_{{1}} {\text{X}}_{{2}} - { 9}.{31} \times {1}0^{{ - {7}}} {\text{X}}_{{1}} {\text{X}}_{{3}} + \, 0.000{\text{1X}}_{{2}} {\text{X}}_{{3}}$$

The accuracy of the model was validated based on the coefficient of determination, pure error, and P-value of lack of fit. The lack of fit of the model provided the p-value of 0.648, confirming that the model was appropriate to define the relationship between process variables and product yield. The fitness of the model was reflected by the value of coefficient of determination of 99.7%, suggesting that the model fitted well with the experimental data. The relatively small pure error of this model was obtained, indicating the reliability of the experimental procedures. Based on all indicators, the model provided high prediction accuracy.

### Catalyst stability

**Figure 6:**
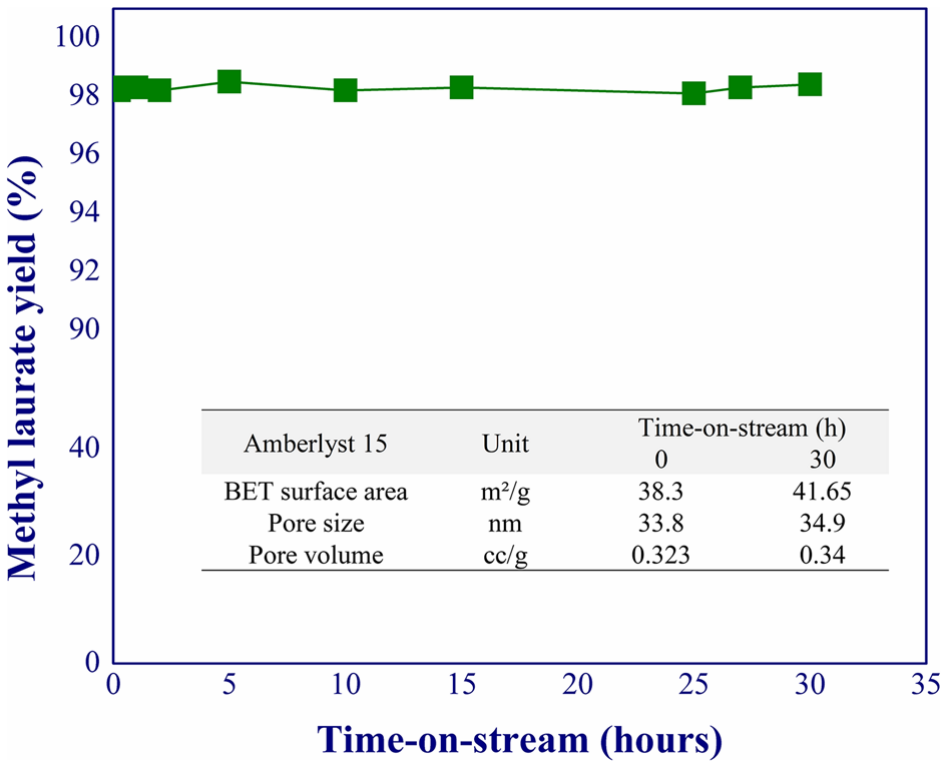
Methyl laurate yield profile during 30 h of time-on-stream; Embed Table: properties of Amberlyst 15.

The catalyst stability was tested under the optimal operating conditions for 30 h of time-on-stream. The catalyst activity in terms of the yield of methyl laurate was used as a monitoring signal to measure the long-term catalyst stability. Figure 6 shows that the yield of methyl laurate was approximately 98% throughout the experimental period. The Brunauer–Emmett–Teller (BET) surface area and pore volume of Amberlyst 15 were not significantly changed after the experiment (see embedded Table in Fig. 6). However, when compared to the stirred-batch process, the losses of BET surface area and pore volume of catalyst resin were observed due to the catalyst attrition after ten cycles of operation^16^. These results suggested that our catalyst was suitable for the continuous production of methyl laurate, offering high yield and selectivity. No catalyst post-treatment and catalyst separation were required compared to the other techniques^10,17^.

### Purification of the product

**Figure 7:**
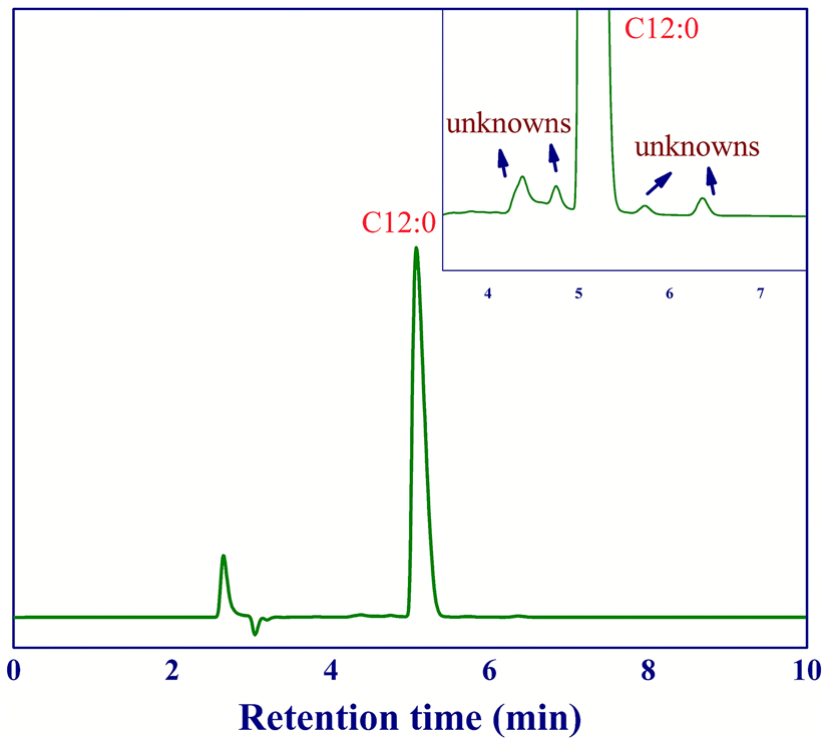
Chromatogram of methyl laurate product obtained at the optimal condition.

Prior to the production of sugar ester by reacting methyl laurate with sucrose, the purification of the product from esterification of lauric acid and methanol, obtained at the optimal conditions, was required in order to remove the large amount of methanol and some water content (by-product). These solvents caused the side reactions during the sugar ester production [see Eqs. (3) to (5)]. Hence, the high purity of methyl laurate was desirable to achieve the high yield of sucrose ester. Product purification via evaporation could be a simple and effective technique to eliminate the solvents (methanol and water). Note that the boiling point at atmospheric pressure of methanol, methyl laurate, and water are 64.7, 267, and 100 °C, respectively. In this work, the product was evaporated under ambient pressure at 105 °C for 6 h. The purity of our purified product was 99%. The impurity in the purified methyl laurate was possibly caused by the impurity of the as-received lauric acid (trace amount of other methyl esters). The chromatogram of the purified product (methyl laurate) is presented in Fig. 7.

### Methyl laurate production performance

Our continuous methyl laurate production was compared to the other processes from the literature based on various indicators, including process type, operating conditions, yield/conversion, and productivity. The results are summarized in Table 2. In batch processes, one of the significant hurdles is the catalyst reusability/stability. Generally, the yield continuously declines after several cycles of operation, limiting its use for large-scale production. Due to the large degree of mixing of batch process, the reasonable yield was achieved by increasing reaction temperature and/or reaction time. Apparently, our work was the first continuous process for the production of methyl laurate from lauric acid. Our continuous process provided high catalyst stability (see Section “Catalyst stability”), possibly improving the cost-competitiveness of the production. Moreover, our proposed method offered the good production performance of 1.4 kg_product_/kg_cat_-h and high yield of methyl laurate (98%) with the relatively short residence time (5 min) and medium reaction temperature (110 °C), when compared to those of the other processes. Note that, the productivity of batch process will generally drop upon the scale-up production. The catalyst reusability will become another problem. Unlike batch process, the large-scale productivity of our process can be similar as that of lab-scale production if the numbering-up assembly is applied. Although large amount of methanol was used, the recycling of ethanol could be easily implemented for practical application. Hence, this process could be used as the first step of the high-throughput and highly selective production of sucrose ester (discussed in the following sections).

**Table 2:** Comparison of methyl laurate production performance with literature.

Process	Reactor type	Catalyst type/amount	Temp (°C)	Time (h)	Ratio (mol/mol)	Yield/conversion (%)	P_0_ (kg_product_/kg_cat_-h)	Ref.
Batch	Batch	Ag_1_(NH_4_)_2_PW_12_O_40_/UiO-66/10 wt%	150	3	15:1	–/75	2.5	^18^
Batch	Batch	Ammonium ferric sulphate/8 wt%	65	1.5	6:1	99/–	8.3	^19^
Batch	Batch	Acid-activated montmorillonites/12 wt%	160	2	12:1	–/95	3.9	^20^
Batch	Batch	Ferric-alginate/16 wt%	65	3	16:1	99/–	2.0	^21^
Batch	Batch	Amberlyst 15/7 wt%	110	3.3	1:1	–/52	NA	^22^
Continuous	Mini fixed-bed	Amberlyst 15/2.7 g	110	0.08	47:1	98/100	1.4	This work

### Sucrose ester production

**Figure 8:**
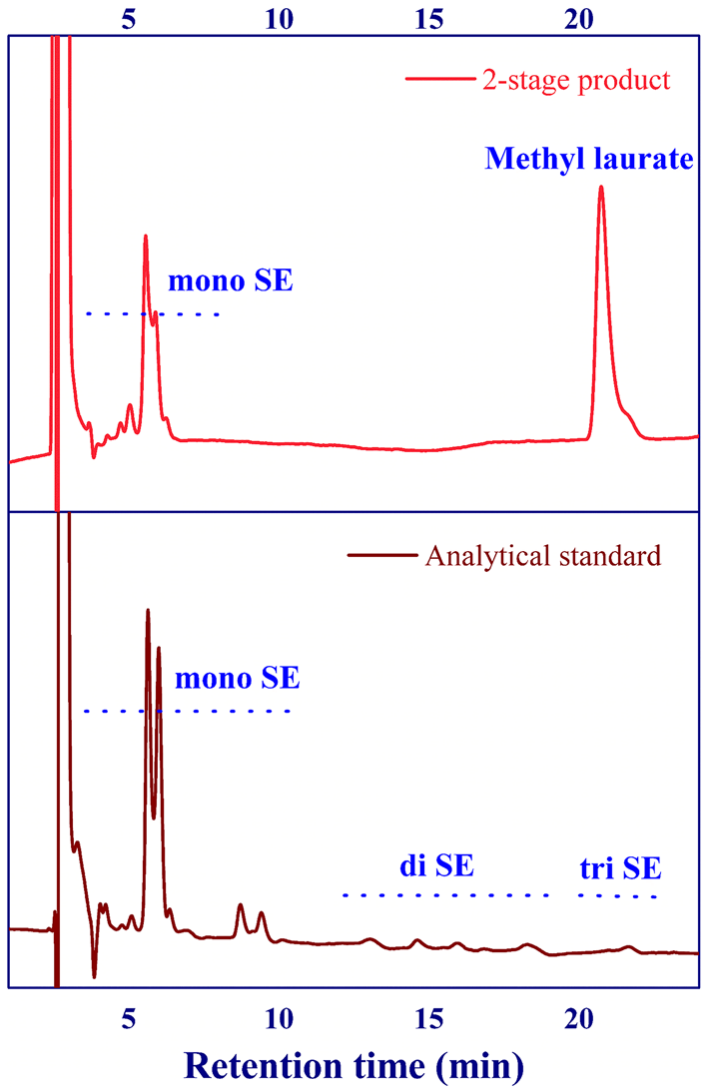
Chromatogram of second stage product and analytical standard of sucrose mono laurate; sucrose monolaurate (mono SE), sucrose dilaurate (di SE), and sucrose trilaurate (tri SE).

In this work, the second stage of the process was demonstrated to validate the feasibility of using a two-stage process for the production of sucrose monolaurate. In this process, the purified methyl laurate obtained from the first stage of the process (Section “Purification of the product”) was used as a raw material to produce sucrose ester via esterification. The operating conditions studied were sucrose concentrations of 5 g/L, molar ratio of sucrose to methyl laurate (S:M ratio) of 0.63:1, reaction temperature of 120 °C, and residence time of 60 min. The results showed that 95% of sucrose monolaurate selectivity (33% of sucrose ester yield) was achieved, confirming the good performance of our proposed sugar ester production through the two-stage process. However, further investigation is necessary to study the influence of operating variables of the second stage process on the sucrose ester yield/selectivity. In addition, the optimization should be performed to achieve the maximum sucrose ester yield/selectivity. The purification of product should also be investigated to achieve the high purity product. All of these works would be undertaken in our future work. The chromatogram of second stage product is shown in Fig. 8. The trace of unknown peaks was not detected on the chromatogram, validating the high selectivity of our proposed process.

The preliminary production performance comparison of our proposed process with literature is summarized in Table 3. The operating conditions, solvent and catalyst types used, yield/selectivity, and productivity were used as performance indicators to initial evaluate the effectiveness of our proposed high-throughput process. Note that, the productivity (P_0_) was modified based on the work of Sasayama et al.^3^. Generally, homogeneous-based batch process has been used for sucrose ester production. Various challenges have been confronted for this conventional process such as scale-up complexity, catalyst separation/purification, and low productivity. Our proposed process offered a continuous process, promising for commercial application. Good production performance (P_0_; 3.0 kg_product_/kg_cat_-h) was obtained compared to the other conventional methods. The sucrose ester yield and productivity of our method could possibly be improved further in our future work. Moreover, the shortened residence time (60 min), low S:M ratio (0.63:1), and ambient pressure could possibly reduce the operating cost, while improving the practicality of the process.

**Table 3:** Comparison of sucrose ester production performance with literature.

Reactor	Raw material	Catalyst type/amount	Temp (°C)	Pressure (kPa)	Time (h)	Ratio (mol/mol)	Yield/selectivity (%)	P_0_ (kg_product_/k_cat_-h)	Ref.
Ultrasonic-assisted batch	Sucrose/methyl laurate	K_2_CO_3_/4 wt%	80	11	2.5	2:1	70/-	5.2	^23^
Ultrasonic-assisted batch	Sucrose/ethyl laurate	K_2_CO_3_/11 wt%	70	11	2	2:1	78/-	9.7	^24^
Mini fixed-bed	Sucrose/methyl laurate	Amberlyst 21/1.7 g	120	101	1	0.63:1	33/95	3.0	This work

## Conclusion

This research introduced a novel two-stage process for the high-throughput production of sucrose monolaurate from lauric acid. In the first stage, the lauric acid was continuously converted into methyl laurate via acid-catalyzed esterification in a mini fixed-bed reactor where the Amberlyst 15 was packed. In the second stage, methyl laurate was reacted with sucrose to continuously produce sucrose methyl laurate via based-catalyzed transesterification with Amberlyst 21 in a mini fixed-bed reactor. In this work, the first stage of the process was mainly focused. All major factors affecting the methyl laurate yield were examined and optimized to achieve the maximum yield. For the first stage, the residence time of 5 min, reaction temperature of 110 °C, and lauric acid concentration of 94 g/L provided the maximum yield of methyl laurate of 98% and the productivity of 1.4 kg_product_/kg_cat_-h. High catalyst stability (Amberlyst 15) was observed with relatively constant yield over 30 h of time-on-stream. The second stage of the process was also demonstrated. The high selectivity of 95% of sucrose monolaurate was achieved. This work is the first step toward pioneering the use of a two-stage process for sucrose monolaurate production with high productivity. The improvement of this proposed production should be considered for practical applications.

## Data Availability

The datasets used and/or analyzed during the current study are available from the corresponding author upon reasonable request.

## References

[1] Szűts A, Szabó Révész P (2012). Sucrose esters as natural surfactants in drug delivery systems: A mini-review. Int. J. Pharm..

[2] Sánchez, M. F. G. *Sucrose Esters Production in a Solvent-Free Reaction System by Transesterification of Sucrose and Fatty Acid Methyl Esters*. https://repositorio.unal.edu.co/handle/unal/76267 (2018).

[3] Sasayama T, Hiromori K, Takahashi A, Shibasaki-Kitakawa N (2021). Process for continuous production of sugar esters of medium-chain fatty acid: Effect of residence time on productivity and scale-up design. J. Food Eng..

[4] Crucesa MA, Ploua FJ, Ferrera M, Bernabéb M, Ballesterosa A (2001). Improved synthesis of sucrose fatty acid monoesters. JAOCS.

[5] Teng Y (2021). Sucrose fatty acid esters: Synthesis, emulsifying capacities, biological activities and structure-property profiles. Crit. Rev. Food Sci. Nutr..

[6] Xie MF, White LV, Banwell MG, Wang Y, Lan P (2021). Solvent-free synthesis of high-purity sucrose fatty acid monoesters and a comparison of their properties with those of their commercial counterparts. ACS Food Sci. Technol..

[7] Fitremann J, Queneau Y, Maître JP, Bouchu A (2007). Co-melting of solid sucrose and multivalent cation soaps for solvent-free synthesis of sucrose esters. Tetrahedron Lett..

[8] Akkarawatkhoosith N, Kaewchada A, Ngamcharussrivichai C, Jaree A (2020). Biodiesel production via interesterification of palm oil and ethyl acetate using ion-exchange resin in a packed-bed reactor. BioEnergy Res..

[9] Tongtummachat T, Akkarawatkhoosith N, Jaree A (2022). Process intensification for 5-hydroxymethylfurfural production from sucrose in a continuous fixed-bed reactor. Chem. Eng. Res. Des..

[10] Boz N, Degirmenbasi N, Kalyon DM (2015). Esterification and transesterification of waste cooking oil over Amberlyst 15 and modified Amberlyst 15 catalysts. Appl. Catal. B..

[11] Su F, Guo Y (2014). Advancements in solid acid catalysts for biodiesel production. Green Chem..

[12] Park JY, Wang ZM, Kim DK, Lee JS (2010). Effects of water on the esterification of free fatty acids by acid catalysts. Renew. Energy.

[13] Chongkhong S, Tongurai C, Chetpattananondh P, Bunyakan C (2007). Biodiesel production by esterification of palm fatty acid distillate. Biomass Bioenergy.

[14] Liu W, Yin P, Zhang J, Tang Q, Qu R (2014). Biodiesel production from esterification of free fatty acid over PA/NaY solid catalyst. Energy Convers. Manag..

[15] Han XX (2013). Efficient and reusable polyoxometalate-based sulfonated ionic liquid catalysts for palmitic acid esterification to biodiesel. Chem. Eng. Sci..

[16] Fu J, Chen L, Lv P, Yang L, Yuan Z (2015). Free fatty acids esterification for biodiesel production using self-synthesized macroporous cation exchange resin as solid acid catalyst. Fuel.

[17] Hykkerud A, Marchetti JM (2016). Esterification of oleic acid with ethanol in the presence of Amberlyst 15. Biomass Bioenergy.

[18] Zhang Q, Yang T, Lei D, Wang J, Zhang Y (2020). Efficient production of biodiesel from esterification of lauric acid catalyzed by ammonium and silver co-doped phosphotungstic acid embedded in a zirconium metal-organic framework nanocomposite. ACS Omega.

[19] Ganesan S, Nadarajah S, Khairuddean M, Teh GB (2019). Studies on lauric acid conversion to methyl ester via catalytic esterification using ammonium ferric sulphate. Renew. Energy.

[20] Zatta L, Ramos PL, Wypych F (2013). Acid-activated montmorillonites as heterogeneous catalysts for the esterification of lauric acid with methanol. Appl. Clay Sci..

[21] Boey PL, Ganesan S, Maniam GP, Khairuddean M, Efendi J (2013). A new heterogeneous acid catalyst for esterification: Optimization using response surface methodology. Energy Convers. Manag..

[22] Banchero M, Gozzelino G (2018). A simple pseudo-homogeneous reversible kinetic model for the esterification of different fatty acids with methanol in the presence of Amberlyst-15. Energies.

[23] Hang FX (2017). Green synthesis of sucrose laurate under different ultrasonic frequencies. Sugar Tech..

[24] Huang DX (2010). Improved synthesis of sucrose fatty acid monoesters under ultrasonic irradiation. Ultrason. Sonochem..

